# Intermittent Atrioventricular Block following Fingolimod Initiation

**DOI:** 10.1155/2014/191305

**Published:** 2014-08-05

**Authors:** E. Gialafos, S. Gerakoulis, A. Grigoriou, V. Haina, C. Kilidireas, E. Stamboulis, E. Andreadou

**Affiliations:** Department of Neurology, Athens National and Kapodistrian University, “Aeginition” Hospital, 11528 Athens, Greece

## Abstract

A 47-year-old female patient with multiple sclerosis (MS) developed symptomatic intermittent 2nd degree atrioventricular block (AVB) of five-hour duration, five hours after the first two doses of fingolimod, that resolved completely. Frequency domain analysis of heart rate variability (HRV) revealed increased parasympathetic activity and decreased sympathetic tone, while modified Ewing tests were suggestive of impaired cardiac sympathetic function. We hypothesize that expression of this particular arrhythmia might be related to autonomic nervous system (ANS) dysfunction due to demyelinating lesions in the upper thoracic spinal cord, possibly augmented by the parasympathetic effect of the drug.

## 1. Background

Fingolimod is an oral sphingosine-1-phosphate (S1P) receptor modulator used for the treatment of relapsing-remitting form of multiple sclerosis (RRMS). Although the drug is safe, certain side effects exist, with cardiac abnormalities, macular edema, and elevation of liver enzymes being the most severe [1, 2]. The mechanism possibly attributed to cardiac abnormalities seems to be the activation of G-protein-coupled inwardly rectifying potassium (GIRK) channels mediated by S1P on atrial myocytes [3]. In clinical trials, fingolimod induces a transient decrease in heart rate, reaching maximum plasma concentration at 4-5 h after the first dose and attenuating over time with continued dosing [4]. In the FIRST study, a 4-month, open-label, phase 3b, multicenter study evaluating mainly cardiac safety during treatment initiation in a real word population with RRMS, palpitations and bradycardia were the most common cardiac abnormalities, with second-degree AVB being rare [2].

We report a female MS patient who developed reversible symptomatic Weckenbach type of AVB after the first two doses of fingolimod and discuss the underlying mechanisms possibly involved in the pathophysiology of this adverse event.

## 2. Case Report

A 47-year-old female with RRMS diagnosed 8 years ago presented with an acute relapse characterized by numbness of the right leg, which started from the toes and ascended up to the right knee. The patient had been discontinuously treated with interferon beta-1b for two separate semesters in the past and had decided to stop treatment on her own 4 years ago, because of flu-like symptoms. The patient is a smoker and her family history revealed MS in her 48-year-old sister and her cousin.

**Figure 1:**
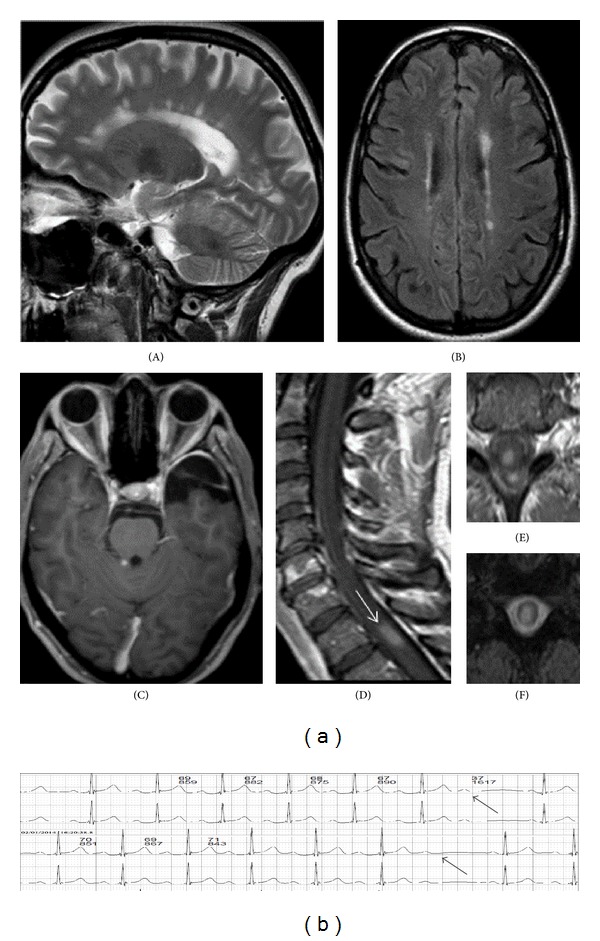
(a) Magnetic resonance imaging of the brain and spinal cord at relapse. (A) Sagittal T2-weighted image showing lesions in the corpus callosum. (B) Axial FLAIR image showing hyperintense lesions in the periventricular white matter of both hemispheres. (C) Axial T1-weighted image showing a gadolinium-enhancing lesion in the right upper and middle cerebellar peduncle. (D) Sagittal T1-weighted image showing a gadolinium-enhancing lesion on thoracic spinal cord at T1-T2 level. ((F) and (E)) Axial T2- and T1-weighted images of the spinal cord showing the extent of the T1-T2 lesion in the axial plane and that of gadolinium enhancement, respectively. (b) ECG Holter strip that shows Weckenbach type 2nd degree AV block 5 hours after initial dose of fingolimod.

Neurological examination revealed impairment of vibration and joint position sense in both lower extremities and decreased sensation of pain and light touch in the right leg. Brain MRI showed demyelinating lesions in T2-weighted images in the cerebral hemispheres and one T1 gadolinium-enhancing lesion located at the right upper and middle cerebellar peduncle (Figure 1(a): (A)–(C)). Spinal cord imaging revealed lesions opposite to C6-C7 and C7-C8 vertebral discs and one gadolinium-enhancing lesion located opposite the T1-T2 vertebra (Figure 1(a): (D)–(F)). The patient was treated with intravenous methylprednisolone 1 g daily for 5 days with clinical improvement. Thereafter, treatment initiation with fingolimod was decided, because the patient denied any injectable medication and there was no contraindication by the prescreening control. The baseline electrocardiogram, cardiac echo, and the 24-ambulatory ECG recording (Holter) did not reveal any abnormalities. The patient started fingolimod with the agreement of the cardiologist, under continuous rhythm monitoring. At treatment initiation her blood pressure was 113/73 mmHg and the heart rate 74 beats/min.

Approximately 5 hours after fingolimod intake, the patient had a feeling of palpitation that had never occurred previously. A Weckenbach type AVB was observed that lasted for 5 hours intermittently and resolved completely (Figure 1(b)). No therapy was applied since it was well tolerated by the patient and the blood pressure was stable (110/60 mmHg). The same event was repeated after the second dose of fingolimod. Thereafter, the drug was discontinued.

**Figure 2:**
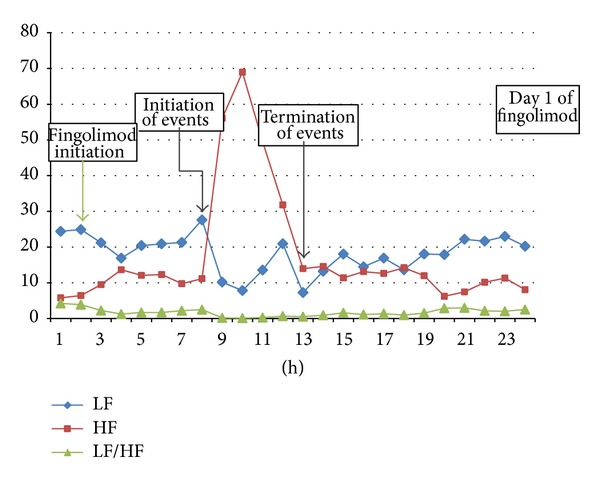
Patient's heart rate variability analysis of the frequency domain during the 1st day of Fingolimod. Low frequency (line with rhombi) implying cardiac sympathetic tone, high frequency (line with rectangles) implying parasympathetic tone, and the ratio of them (line with triangles) implying ANS abnormality.

Subsequently, it was decided to evaluate the patient's autonomic nervous system function by reviewing heart rate variability (HRV) from Holter's R-R intervals and applying modified Ewing's tests, namely, orthostatic, sustained handgrip, and deep breathing test. Frequency domain analysis of HRV showed an increased parasympathetic activity and a decreased sympathetic tone implying ANS abnormalities (Figure 2). In Ewing tests, whereas the patient reacted normally to both orthostatic (heart rate and blood pressure response) and deep breathing tests, she could not increase diastolic blood pressure during sustained handgrip, suggesting impaired cardiac sympathetic activity.

## 3. Discussion

Although fingolimod is generally well tolerated, certain side effects can be observed, with cardiac conduction abnormalities being the most urgent to be recognized [5–7]. Our patient, who had apparently normal baseline 24 hour ECG recording but autonomic nervous system dysfunction, developed symptomatic intermittent 2nd degree AVB of five hours duration, during the first two doses of fingolimod. Remarkably, both episodes started five hours after medication intake and resolved spontaneously.

Atrioventricular node is highly innervated by fibers of the left vagus and cardiac nerve, which respond to parasympathetic and sympathetic nervous system firing, respectively [8]. The nodal function depends among others on their balance; therefore disturbance of this equilibrium results in overactivity of the dominant system. Our patient exhibited intermittently 2nd degree AVB with increased parasympathetic activity 5 hours after treatment initiation, which is in agreement with previous reports [2]. The observation of a parasympathetic-like effect of fingolimod might be due to its binding of S1P receptors located in cardiac tissue [3]. This transient effect is related to a short, S1P1-dependent activation of the G protein-gated potassium channel IKAch in atrial myocytes, prior to internalization and/or desensitization of the S1P1 receptors by the drug [4]. In our case, heart rate analysis revealed inability of the sympathetic system to compensate this enormous increase of the parasympathetic activity. To our knowledge, this remarkable finding has not been previously reported in the literature. Moreover, the patient failed to increase the diastolic blood pressure to sustained handgrip (>16 mmHg after 3 minutes of maximum voluntary exercise) that is an additional sign of partial sympathetic failure.

This decreased sympathetic activity might be correlated with the spinal cord lesions detected on MRI of the patient at the inferior cervical and superior thoracic level (T1-T2). The cardiac sympathetic preganglionic fibers derive from neurons in the lateral horn of the upper four-to-five thoracic segments of the spinal cord. These preganglionic fibers project to the cervical and upper thoracic ganglia of the sympathetic chain and provide the postganglionic neurons for the innervation of the heart [8]. Indeed upper thoracic spinal cord lesions have been previously associated with myocardial ischemia and arrhythmias [9, 10].

Patients without preexisting cardiovascular disorders and those not taking heart-rate-lowering or QT-prolonging drugs are eligible to receive fingolimod. Until now, only 6-hour monitoring period was considered sufficient. However our finding and other reports [5–7] stress the importance for extensive monitoring period in certain cases. Given that structural lesions in brain stem and/or thoracic spinal cord might interfere with ANS function, application of Ewing tests prior to treatment initiation in these cases might be useful in predicting possible arrhythmias.
